# (Neg)Entropic Scenarios Affecting the Wicked Design Spaces of Knowledge Management Systems

**DOI:** 10.3390/e22020169

**Published:** 2020-02-01

**Authors:** Ulrich Schmitt

**Affiliations:** Business School, University of Stellenbosch, Bellville Campus, Carl Cronje Dr., Bellville, Cape Town 7530, South Africa; schmitt@knowcations.org; Tel.: +267-7211-3660

**Keywords:** knowledge management, personal knowledge management, knowledge management systems, Popper’s worlds, entropy, granularity, systems thinking

## Abstract

The envisioned embracing of thriving knowledge societies is increasingly compromised by threatening perceptions of information overload, attention poverty, opportunity divides, and career fears. This paper traces the roots of these symptoms back to causes of information entropy and structural holes, invisible private and undiscoverable public knowledge which characterize the sad state of our current knowledge management and creation practices. As part of an ongoing design science research and prototyping project, the article’s (neg)entropic perspectives complement a succession of prior multi-disciplinary publications. Looking forward, it proposes a novel decentralized generative knowledge management approach that prioritizes the capacity development of autonomous individual knowledge workers not at the expense of traditional organizational knowledge management systems but as a viable means to foster their fruitful co-evolution. The article, thus, informs relevant stakeholders about the current unsustainable status quo inhibiting knowledge workers; it presents viable remedial options (as a prerequisite for creating the respective future generative Knowledge Management (KM) reality) to afford a sustainable solution with the generative potential to evolve into a prospective general-purpose technology.

## 1. Entropy, Syntropy, and Negentropy in Recent Knowledge Creation and Management Contexts

The concept of ‘Entropy’, defining the degree of disorder in the second law of thermodynamics, also serves as a metaphor for the irreversibility of knowledge transformation and for today’s increasingly agile knowledge societies driven by flatter management hierarchies in need of higher vertical and horizontal knowledge transfer intensities. The term ‘Syntropy’ has likewise been repurposed to emphasize the managerial necessities for the continuous “upgrading of organizational energy, knowledge and intelligence through increasing alignment, or integration of all resources and capabilities” [1] (p. 5) towards anticipated potentials and purpose.

Organized human minds may, hence, create negative entropy (or ‘Negentropy’) but, in the process, need to cope with vast, complex, conflicting, inconsistent, and fragmented information loads. Fueled by growing mobile internet access and social networks, the traditional information scarcity (characterized by few knowledge sources/channels associated with high costs) has recently transformed into today’s never-before experienced information abundance whose dynamic expansion and evolution are “threatening the finite attention individuals’ cognitive capabilities are able to master” [2,3] (p. 10).

Recent systematic literature reviews (2004–2017) have focused, therefore, on information overloads affecting individuals (due to the diversity of media, content, sources, and channels) and institutions (due to impediments to workflows, productivity, and decision-making). Suggested remedies include advancing information literacy and hubs, the rating of content relevance or filters, posting of guidelines and experts’ skill profiles, and improving system design. However, the papers reviewed tend to focus on single managerial or technological aspects without engaging with their interactions and with potential systemic methodological or technical resolutions [4,5].

This article, in contrast, takes a holistic view at this ill-defined ‘wicked’ problem space with its “incomplete, contradictory, and changing requirements and complex interdependencies” where the reasoning and information needed to understand the challenges depends upon one’s idea or concept for solving them [6].

As part of an on-going design science research (DSR) and prototyping project, it complements prior publications in developing a Personal Knowledge Management (KM, PKM) System (KMS, PKMS) and in aiming for theory effectiveness, a DSR principle expecting system designs to be incrementally and iteratively designed to be purposeful in terms of their utility (largely a matter of content) and their communication (largely a question of presentation to an audience) [7,8]. The notion also implies that relevant methodologies and practices need to be scrutinized to potentially integrate them for continuous thorough design evaluation and knowledge dissemination. Thus, the objective is to further validate the PKMS’s utility and theory effectiveness by focusing on current entropic KM unsustainabilities and on the envisaged negentropic affordances of the proposed networked PKMS devices.

The next sections further explore the sources of entropic potentials (Section 2), the recent changes causing their expansion (Section 3 and Section 4), the need to shift the current document-centric paradigm to a memetic storage approach (Section 5), the envisaged benefits of re-inventing the knowledge-creating model in this way (Section 6), the negentropic consequences of related value-adding services (Section 7), and the rationale from a meta-point-of-view as well as from a nano-micro-meso-macro-perspective of transforming entropic to negentropic states (Section 8 and Section 9).

## 2. Entropic Potentials in the Context of Personal and Institutional Absorptive Capacities

Wiig emphasizes that organizational viabilities and advances depend on innumerable small ‘nano-actions’ by individuals (knowledge workers) which govern, if effectively combined, the institutional (knowledge economy) and societal performances (knowledge society) [9].

In the underlying transitional contexts, an individual’s maximum explicable knowledge and tacit knowhow carrying capacity has been defined as one ‘personbyte’. Any ambition or demand exceeding this unit requires organizational interventions to deal with individual learning, experience, talent, and attentiveness restrictions as well as of collective institutional limitations (including dicing and distributing that knowhow to fit its personbyte equivalents) [10] (Hidalgo, 2015, pp. 81–82). Institutions in this context represent “snapshots of a subset of the ideational field that persevere while the network itself continues to fluctuate” [11] (p. 2) exemplified by teams, social networks, firms, cities, regions, economies, or countries.

As growing institutions face escalating transactional interventions and expenditures, limits may be reached (institutional maximum knowledge and knowhow carrying capacity or ‘firmbyte’) where external collaboration becomes less costly and, hence, preferable. While cheaper links afford larger network, too costly links trigger fragmentation. The innovation and roll-out needs of today’s complex products, hence, may require the networking of institutions’ limited diverse knowledge carrying capacities (firmbytes) and the rigorous controlling of the associated additional transaction costs [10] (pp. 89, 91–93).

Distributing large accumulated knowledge volumes by repackaging and disbursing them via firmbytes and personbytes allows for nested structures where networks of neurons become people (to contribute individual ‘nano’-crystallized imaginations) which may become nodes in an institutional network, and which may scale as nodes in a network of institutions. Due to the inherent relational complexities, such progressing knowledgeable networks are not only hard to form and sustain but even harder to move to or replicate in other locations and, thus, are geographically circumscribed [10] (pp. 106–107, 129).

Integrating information loads into carrying capacities depends on specific practiced routines (local instantiations of theoretical micro-foundations of absorptive capacity (AC) like procedures, habits, norms, or rules) related to generalized meta-routines meeting interdependent organizational contexts and envisaged innovation performances as, for example [12]:

- Internal AC meta-routines for managing variation, selecting, sharing, reflecting, updating, and replicating as well as some form of comparison to the external environment for managing adaptive tension (e.g., reference groups, benchmarking).
- External AC meta-routines for identifying, recognizing, and learning from valuable externally generated knowledge in collaboration with stakeholders, as well as transferring external knowledge back by linking it to in-house capabilities.

This routine-based absorptive capacity theory/model facilitates the development of organizational (negentropic) processes and capabilities for acquiring, exploring, creating, transforming, exploiting, and assimilating knowledge as well as “finding complementarities between configurations of meta-routines that leverage the effectiveness of individual practiced routines” [12] (pp. 94–95). Its micro-foundations/meta-routines AC approach was also successfully applied as the basis for conducting a concept-centric systematic literature review, for developing an integrated framework for information and knowledge management systems (KMS), and for researching six small and medium enterprises (SME) in the information technology sector [13,14].

The results of the latter emphasize the capital expenditures, resource allocations, and time/effort consumptions required. They refer to the inherent risks of lacking the agility to record, screen, absorb, transfer, share, exploit, deploy, update, react to, reflect and/or give feedback on new knowledge in time as well as of inadequate archiving, documenting, quality assuring, or mentoring processes, of inefficient assistive technologies (e.g., document repositories, training and KM systems), policies (e.g., empowerment and motivating incentives), and performance or impact metrics [14] (pp. 253, 271, 282–284). These respective entropic potentials do not even include the growth-related challenges SMEs and large institutions face [15], nor the negative consequences of (un)discoverable public and private knowledge to be referred to later.

## 3. Popper’s Three World Perspective and Its Role in a Novel PKMS Conceptualization

The quantity and quality of ‘nano’ actions and contributions depend on peoples’ individual competences, skills, and intellectual, social, emotional, and structural capitals [9] whose presence or absence can either be a source of productive absorptive capacities (ability to recognize, assimilate, and apply new valuable information) or cause of destructive opportunity divides [16].

**Figure 1:**
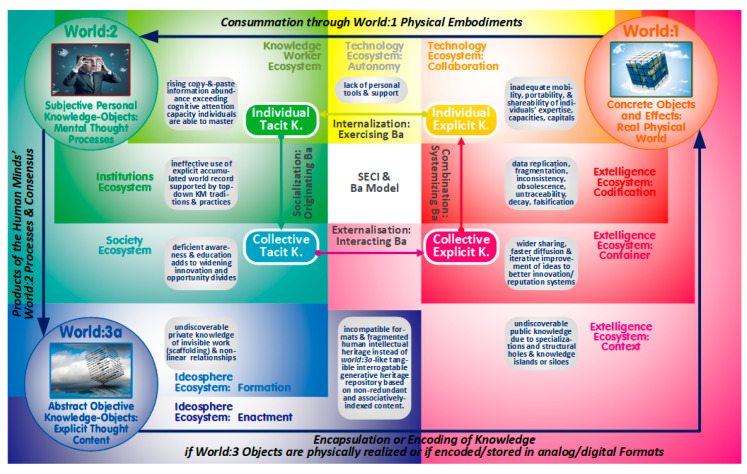
Popper’s Three Worlds aligned to Ten Digital Ecosystems, and Nonaka’s Ba and SECI (socializing, externalizing, combining, and internalizing) Model.

Popper introduced a three-world-metaphor (Figure 1) to explicate the underlying contextual knowledge creation processes [17]. The differentiation between the physical world:1 (concrete knowledge objects and their relationships and effects) and the human minds’ world:2 (subjective personal knowledge objects) marks an approach which has subsequently been adapted to explain wealth creation as a result of co-evolving physical and social technologies [18] and knowledge creation as a result of co-evolving technological and social innovations and design spaces [19,20]. While these adapted notions task a third entity with being a co-evolutionary driver (plans, perceptions, or nurturing of leaders and innovators), Popper’s world:3a was mainly meant to serve his conviction that thoughts - in order to be shared or critiqued—need to be explicated, so that the formulated content stands objectively on its own (independent of its creators and able to be judged on its own merit).

Accordingly, Popper’s world:3a accumulates crystallized imaginations of the world:2’s human minds as abstract explicated master copies (of original and/or mutated knowledge objects) irrespective of their concrete embodiment(s) as physical world:1 object(s). By complementing the subjective tacit world:2 mental personal thought processes with the abstract explicit world:3a objective content perspective, Popper divorces the creator from his/her message which—to become accessible and elicit impact—still needs to be resourcefully combined and physically encapsulated (or encoded) in concrete physical objects to transform the concrete world:1 environment (or other world:2 minds) in order to potentially yield further world:3a objects and/or relationships [17]. World:3a, thus, is just a philosophical construct bridging the minds and physical worlds only metaphorically.

A recent publication benchmarked twelve dynamic knowledge creation models against each other (including Nonaka’s SECI & Ba Spiral, Boisot’s Information Space, Wierzbicki’s & Nakamori’s Seven Waterfalls Model, and Pirolli’s & Card’s Foraging and Sensemaking Loops). In recognizing their complementary nature, an integrated three-dimensional dynamic ‘public-transport-like’ map was created [21]. Their common feature is that all these models have been introduced during the former period of information scarcity alluded to. In such an environment, redundant information can be seen as a blessing (to find needed knowledge more easily), and, so, the few isolated warnings and remedies proposed (e.g., Bush’s ‘Memex’ [22], Nelson’s ‘Xanadu’ [23], or Simon’s attention management [2]) have been graciously ignored so far.

For the purpose of this article, the theory of organizational dynamic knowledge creation (as one of the most widely cited and renowned KM theories [24]) has been adopted. Its SECI model (center of Figure 1 displaying its socializing, externalizing, combining, and internalizing workflow cycle) also adopts the social (tacit knowledge) and physical (explicit knowledge) differentiation but subdivides the knowledge types/stocks further into respective individual and collective segments. The transforming third role is carried out within distinct enabling spaces parallel to the SECI workflows (called originating, exercising, systemizing, and interacting ba) in the anti-clockwise direction depicted [25]. Its stocks and flows are aligned to eight digital ecosystems related to world:1 and world:2. Largely unconcerned with entropic redundancy considerations (exemplified in Figure 1 (bottom) to be detailed later), the SECI model has been depicted isolated from two additional ecosystems (ideosphere) linked to world:3a.

As an extension to the SECI’s KM-horizon, the three Popperian worlds [17] have proven useful for the initial analysis as well as the subsequent meta-conceptualization of the novel generative PKMS to be detailed further. Their related ten digital ecosystems (based on the ideas of Gibson [26] and Briscoe [27] and depicted in Figure 1 in circular fashion corresponding to a color wheel) allow for distinguishing spaces based on particular objectives, structures, agents, fixations, affordances, and dynamics which fully align to a diversity of generative system attributes and functionalities [8,28]. The world:3 notion also affords conceptualizing the negentropic future of an evolving rational human intellectual heritage to combat the current distracting entropic outcomes of non-curated and unsustainable knowledge diffusion.

## 4. Outdated Book-Age Diffusion Paradigm as a Cause of Escalating Entropies

Unfortunately, world:3a, is currently just an abstract metaphor and neither accessible nor interrogatable. Neglected by the externalizing workflows of blindsided bygone KM models, the envisaged knowledge societies continue to struggle with the outdated ways of knowledge object diffusion and their entropic consequences, exemplified by replication, fragmentation, inconsistency, obsolescence, untraceability, corruption, decay, or falsehood.

One of the key culprits is the “over-simplistic modelling of digital documents as monolithic blocks of linear content, with a lack of structural semantics [which is] unnecessarily replicating content via copy and paste operations, instead of digitally embedding and reusing parts of digital documents via structural references” [29] (p. 391). The causes and effects of this outdated book-age approach have been further pointed out by a world:3-based small/big-theories/knowledge-scheme [7,30] which links the world:1’s collective tacit with the world:2’s collective explicit knowledge types. From this perspective:

- An individual knowledge worker applies learnt knowledge to his/her own ideas (to be referred to in the following as memes) to crystallizes and explicates them over time (in acts of cumulative synthesis [31]) which occupies a kind of personal world:3a ‘pigeon hole’ containing an up-to-date aggregated variety of his/her abstract, non-linear, partially connected, meme explications.
- This ‘pigeon hole’ is only accessible and interrogatable via the individual’s own memories and/or knowledge cues (defined as “external reminders about previously experienced internal knowledge” which may be “any kind of symbol, pattern or artefact, created with the intent to be used by its creator, to re-evoke a previously experienced mental state, when used” [32] (pp. 3, 21)).
- Whenever an already explicated and accessible meme from the ‘pigeon hole’s’ historic content is paraphrased, re-versioned, or annotated (or an original meme is recorded), novel world:3 content is created. If others’ explicit world:1 memes are directly or indirectly quoted, scholarly practice requires citing the original source by referencing their world:2 authoring or editing minds and physical world:*1* container (e.g., document, file, web page).
- This personal world:3 ‘pigeon hole’ contains the objective linguistically formulated thought content in the form of abstract explicit knowledge objects and represents—in terms of this argument—the individual’s holistic ‘big-TK’ (theory/knowledge). As a non-physical imaginary virtual construct, it is neither accessible nor interrogatable by others. Accordingly, it may only be effectively disseminated via resourcefully combined subsets of ‘small-TKs’ in a variety of world:1 physical/digital artefacts and knowledge assets (defined as “nonphysical claims to future value or benefits” [33] (p. 335)) as, for example, research papers, conference presentations, chapters, reports, or prototypes.
- The world:3a-to-1-transitional ‘small-TK’ instantiations are, however, susceptible to gaps, disconnects, overlaps, and sporadic modifications “due to, for example, page restrictions, diverse target audiences, confidentiality considerations, project progress updates, error retractions, sponsoring agreements, and partial or extended re-publications“ as well as reviewers’ demands, editing or curating interventions, datedness, erroneous quoting or citing, incorrect copies, and falsifications [30].

While the massification of higher education and expanding academic publish-or-perish practices are amplifying the number of authors (with the authority of traditional physical filters and curating authorities declining), voicing of any perceptions has eased with today’s one-click-online-publication options. These dynamic settings, however, have also provided fertile ground for unproductive entropic side-effects (see rounded grey rectangles in Figure 1):

1. The over-simplistic copy-and-paste digital document creation alluded to contributes progressively to the share of information entropy to be perceived as overload. As discoverable entropic public knowledge, it is threatening the finite attention and carrying capacities referred to in the introduction. This unsustainable situation is aggravated by the lack of personal tools and mentoring which also inhibits the mobility, portability, and shareability of individuals’ expertise, capacities, and capitals.
2. Adding to the woes of discoverable knowledge constraints are current online and publishing realities which inhibit engagement in a wider sharing, faster diffusion, and more rapid iterative improvement of ideas, sources, data, work-in-progress, preprints, and/or code and, consequently, are stifling innovation as well as academic reference/reputation systems [34].
3. The non-redundant explicated world:3 knowledge also expands with academic and technological advances but is, at the same time, weakened by proliferating and growing “structural holes” [35], referring to the potentially beneficial but unrecorded ties between knowledge clusters ranging from memes, over to approaches, to specializations and disciplines (the theories of organizational learning and knowledge creation, for example, “have been pursued as independent themes for almost two decades” [36] (p. 113). The lack of connectivity contributes to undiscoverable public knowledge (islands and silos) and inhibits methodological approaches to better tackle complex transdisciplinary ‘wicked’ problem spaces.
4. A further weakness is today’s ineffective utilization of the explicit accumulated world record supported by top-down KM traditions. Stewart and Cohen [37] coined the term ‘extelligence’ to position it as the externally stored counterpart to the intelligence of the human brain/mind tasked with understanding; together they are driving each other in a complicit process of accelerating interactive co-evolution.
5. Due to the deficient awareness (and respective educational interventions) regarding the status quo, personbytes and firmbytes are severely constrained as extelligence only generates competitive advantage if it is accessible and augmentable by individuals who know how [37]. As a result, societies are facing widening innovation and opportunity divides.
6. Current reviewing and publishing practices also prevent the sharing of “magnitudes of invisible work” (defined as the “gap between formal representations, including publications, and unreported ‘back stage’ work” [38] (pp. 606–607) termed by Bush [22] as the “scaffolding” of research output. As undiscoverable private content knowledge, others are forced to re-spend the energy and to start over.
7. Moreover, today’s digital authoring still compels us to provide linear accounts (digital documents following the outdated book-age paradigm) of nonlinear realities (Popperian world:3 as well as real world) preventing the effective sharing of knowledge already understood in holistic transdisciplinary ways. As undiscoverable private non-linear relational knowledge, this cause prevents benefitting from knowable and sharable nonlinear content and contexts.
8. Non-standardized incompatible formats continue to underpin fragmented records of the human intellectual heritage instead of unlocking access as well as collaboration capabilities by instantiating a world:3a-like tangible interrogatable generative heritage repository based on non-redundant and associatively indexed content.

Table 1 summarizes the causes and effects to emphasize the deteriorating dilemma for both the current systemic leadership (tasked with creating enabling ba/spaces) as well as for individuals’ absorptive and creative capacities (tasked with effectively contributing to the world:1 extelligence).

## 5. Shifting Paradigm from Document-Centricity to a Tangible Memetic Popperian Third World

To update the rationale for the PKMS remedy, a prior article utilized the nine-step SVIDT methodology (strengths, vulnerability, and intervention assessment related to digital threats) to reverse engineer the envisaged PKMS affordances back to the underlying motivations [30,39]. In an ensuing publication, the psycho-social notion of generativity was employed to embed the rationale within a holistic systemic KM review and to sketch a future trajectory of KM systems [40]. This article’s focus on the entropic concerns is the logical next step and will be followed up by crafting a desirable sustainability vision for the novel, potentially disruptive PKMS technology (paper currently in progress).

The foundational base of all these considerations is a shift from the current traditional book-age and document-centric storage paradigm to a digital-age, Popperian world:3b, memetic paradigm.

Memes were originally described by Dawkins [41] as units of cultural transmission or imitation (e.g., ideas, tunes, catch-phrases, skills, technologies). They are (cognitive) replicating information-structures that evolve over time—analogous to genes—through a Darwinian process of variation, selection, and transmission with their longevity being determined by their environment. As a metaphor of ‘living organisms’ (as promoted by Memetics), they afford a useful conceptual scheme for knowledge and ideas whose survival depends on enduring in their medium of occupation and on the endurance of the medium itself.

The PKMS’s re-interpretation of memes share a common digital structure and their relations are either integrated in it or represented by other discrete memes (self-referential). Their format—as compared to the current document-centric storage practice—is standardized. As a result, any actor’s personal knowledge base as well as any artefact at any stage in the progressing value chain (meme, memeplex, knowledge or learning asset) represent a unique subset of the accumulated tangible Popperian world:3b flat-file knowledge repository.

Fortunately, the living-organisms-metaphor of memes is also offering synergetic advantages for communicating the Popperian and SECI flow descriptions (Figure 1, center): For survival, memes “either need to be encoded in inanimate durable world:1 vectors (such as buildings, machines, products, software, storage devices, books, great art, or major myths) spreading at times unchanged for millennia, or to succeed in competing for a living host’s world:2 attention span (such as people, teams, corporations, or economies) to be [subjectively and tacitly] memorized (internalization) until forgotten, codified (externalization) in further concrete world:1 objects [(via objective abstract world:3a objects)] or spread by the spoken word to other hosts’ world:2 brains (socialization) with the potential to mutate into new variants or form symbiotic relationships (combination) with other memes (memeplexes) to mutually support each other’s fitness and to replicate together” [30].

## 6. Utilizing the Memes’ Higher Granularity for Re-Inventing the Knowledge-Creating Model

**Figure 2:**
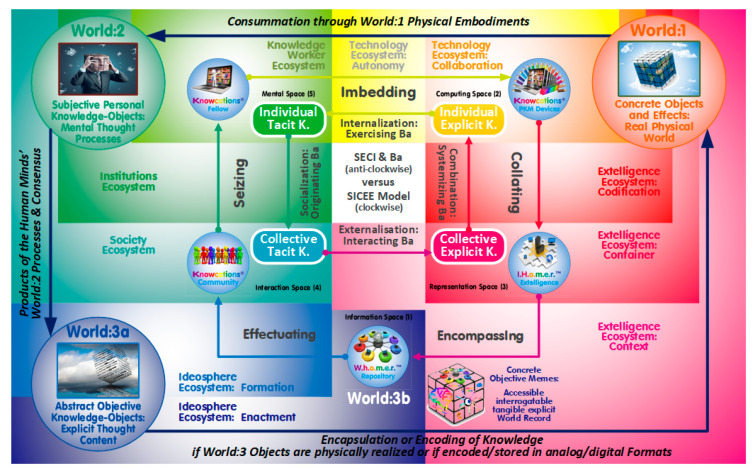
Popper’s Three Worlds aligned to Six Digital Ecosystems, and Nonaka’s SECI/Ba Model, and the Conceptualization of a Modified SICEE Workflow as Part of a Novel Personal KM System.

Figure 2 reconfigures its predecessor by replacing the rounded rectangles (side-effects) with the workflows model of the novel PKMS configuration (aligned to the Popperian worlds, ecosystems, and SECI cycle presented earlier).

### 6.1. The Framework of the Ten Digital Ecosystems

By differentiating society, institutions, and individual knowledge workers as three distinctive world:2 ecosystems (top-left greenish sections, Figure 2), the SICEE model incorporates the hierarchy of personbyte, firmbyte, and economy alluded to. The world:1 extelligence (bottom-right redish sections, Figure 2) is structured likewise with the accumulated transdisciplinary knowledge heritage (context) further differentiated in knowledge and learning assets (container) constructed from memes and memeplexes (codification). This explicit or encapsulated ‘soft’ extelligence is complemented by their autonomy-and-collaboration-related technological ‘hard’ artefacts (top-middle yellow/orange sections) resulting in a five-fold segmentation of Popper’s world:1 in support of Hidalgo’s emphasis of products as amplifiers of human minds [10]. The ecosystems’ alignment corresponds to the anti-clockwise SECI cycle as well as the clockwise SICEE sequence which also includes the formation-and enactment-related world:3 ideosphere ecosystems with their entropy-related concerns (bottom-right blueish sections, Figure 2).

### 6.2. The SECI Versus the SICEE Cycle Versus the Sensemaking Loop Model for Intelligence Analysis

Commencing with the individual tacit knowledge stock, the SECI’s model follows the anti-clockwise cycle depicted (socializing-externalizing-combining-internalizing). While each of the eight SECI flows and stocks correspond to eight of the ten PKMS flows and stocks/structures/spaces embedded in their respective ecosystems, a further space and flow are complementing the SECI model having been placed in the world:3ab-related ideosphere ecosystems. The novel PKMS concept, thus, reverses the SECI order in the clockwise SICE(E) sequence which retained the first letters of the SECI acronym although with modified semantics (seizing-imbedding-collating-encompassing-effectuating) to emphasize the inherent synergies between the two cycles.

**Figure 3:**
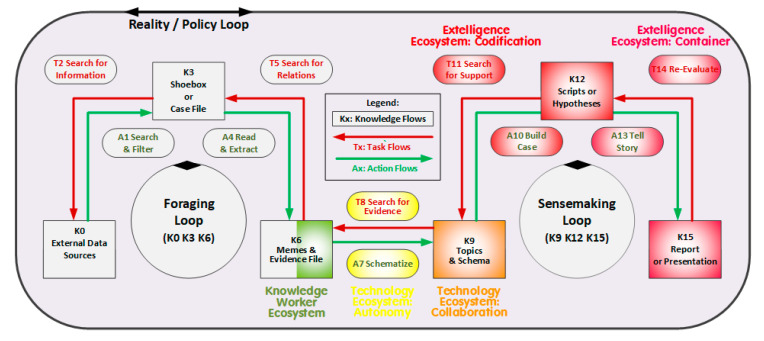
Pirolli’s and Card’s Notional Model of the Sensemaking Loop for Intelligence Analysis [42].

It also partially integrates a further knowledge creation model included in three-dimensional map mentioned earlier [21]: Pirolli and Card’s cognitive-task-related notional model of the sensemaking loop for intelligence analysis [42] which focusses on individuals’ work by integrating a foraging and sensemaking loop under the umbrella of a reality/policy loop. It correlates with five of the ecosystems as shown in Figure 3 and as explicated in the following sections.

#### 6.2.1. The Foraging Loop

From the outset of the real-world processes, the foraging loop (or reality part of the reality/policy loop) presents a shared basis with the SICEE model:

- External data sources [Knowledge K0] are identified, verified, contacted, and evaluated [Action A0] according to (initial or subsequently adapted) information needs [Task T2] and interrogated during field and desk research. The data collected is filtered for relevance [A1] and temporarily stored in a physical ‘shoebox’ or digital case file [K3].
- The case file [K3] is accessed for further screening, reading, sorting, and extraction [A4]. Experiences gained allow for re-assessing and revising related sources and content [T5], for adjusting evidence [K6], or to support additional evidence requirements [T8] in order to continually improve the value of information subsets passed on from the shoebox [K3] to the evidence or memes file [K6] (Knowledge Worker Ecosystem).
- What may be captured during project work in the individual’s PKMS repository depends on the evidence gathered [K6], on the schematizing to be covered (e.g., classifying, contextualizing, interpreting, annotating, summarizing, or formatting) [A7], and on the further needs established [T8] (Imbedding Space based on Autonomous Technology Ecosystem).

#### 6.2.2. The Sensemaking Loop

The sensemaking loop (or policy part of the reality/policy loop) includes the workflows in the Pirolli model where the intelligence analyst scrutinizes the evidence and prepares for his/her findings. The relevant workflows can be fully supported by the SICEE model:

- Captured content is stored in the topics/schema or memeplexes file [K9] and may be linked on an on-going basis to the established/emerging schemas [A7] which allow analysts to structure the content for later synthesis and communication (Collaborative Technology Ecosystem).
- Once sufficient content is available, the analyst/author may start building his/her case [A10] which may need some support [T11] but, if finalized after some potential re-evaluation [T14], results in, for example, scripts, hypotheses, conclusions, or new meanings [K12] (Collating Space within Extelligence Codification Ecosystem).
- The different content sections may be cumulatively synthesized to tell the story as knowledge or learning assets [A13] and, after further re-evaluations [A14), are to be shared, presented, or published via different media [K15] (Extelligence Container Ecosystem).
- Utilizing a PKMS requires a further decision on what part of any newly created or prior confidential personal work to voluntarily share with the PKMS community. The memes chosen may significantly outstrip any document shared or published traditionally. Due to the links and associated knowledge objects, the PKMS community may receive an information-richer content with the negentropic potential to limit invisible work and structural holes and to afford better online diffusion options and non-linear publishing (Table 1) (encompassing space within extelligence context ecosystem). Confidential meme categories further allow monitoring projects-in-progress [T2,5,8,11,14] which contain: Forethoughts focusing on longer-term objectives, plans, and related responses; Intentions focusing on shorter-term tasks and diaries, and Evaluations focusing on feedbacks and personal assets and reflections.

Sensemaking in the context of PKMS implies active information processing for understanding (instead of achieving a specific world state) and “requires learning about new domains, solving ill-structured problems, acquiring situation awareness, and participating in social exchanges of knowledge” [43]. The task-related feedbacks [T2,5,8,11,14] may signal the unavailability or unaffordability of sources, facts, theories, or methods and trigger remedial actions as, for example, training, expert advice, or the self-development of tools. The progression of content [K0,3,6,9,12,15] may involve aiming for its flexible re-representation and re-purposing to also contribute to other sensemaking or authoring activities. Accordingly, generic tool repositories (also termed ‘boundary objects’) containing, for example, standards, templates, methods, flow charts, heuristics, benchmarking criteria, or directories may be set up to facilitate experience management for better assisting the structured activities [A1,4,7,10,13].

#### 6.2.3. The Popperian World:3b Creating and Curating Process

Once the shared updates from the individuals’ decentralized local meme capturing or authoring sessions with their interlinked new memes are uploaded to the cloud-based centralized PKMS, they are subjected to a range of curation processes (Ideosphere Enactment Ecosystem):

- Together with any global content updates contributed by the PKMS own or partner services, the updates are aggregated and immersed within the cumulatively synthesized historic knowledge base, termed World Heritage of MEmes Repository (WHOMER). Although its structure resembles a complex entity-relationship-model, any meme or connection also just represents a distinctly structured generic record which can be intercompared by the centralized services to allow for vetting to identify any duplicates.
- In such a case, identical memes from different sources are merged while their relationships with diverse meme sets and usage histories are fused to preserve all information. A reference record of every meme shared is also kept, even if it might be blocked from dissemination due to, for example, legal, ethical, or falsification reasons. Any identical meme uploaded in the future is, hence, identifiable to trigger appropriate actions.
- The consolidation and curation of this interrelated, associatively indexed, multi-disciplinary content is envisaged to steadily mature—with a growing community and meme base—into a single unified digital knowledge repository representing the tangible interrogatable equivalent (world:3b) of the philosophical notion of Popper’s abstract intangible inaccessible third world:3a.
- All the interventions alluded to, so far, aim for the associative integrity of the WHOMER knowledge base (analogous to the relational integrity of relational databases). Once this state is secured, the updated repository can be made accessible to the PKMS community.

The last workflow in the SICEE cycle hosts additional centralized value adding WHOMER services complementary to the SECI model to address further entropic issues addressed in Section 7 (Effectuating Space within Ideosphere Formation Ecosystem).

#### 6.2.4. Feeding Back the World:3b Updates to the Decentralized Devices of the PKMS Community

The PKMS decentralized devices afford integrating their relevant updated world:3b subset within their historical personal repositories. Additional records from the world:3b WHOMER knowledge base may also be accessed via search functions or by following associative indexes previously not traced. The memes encountered may be integrated in the personal knowledge base, just as any meme captured manually via the foraging loop alluded to (Section 6.2.1).

In addition to capturing external or accessing internal memes, users may create content personally by creating new memes/relations or by altering the attributes of existing memes in any three-way combination: changing a meme’s symbols and codification (revising), its application and container/asset (redeploying), and/or its meaning and context (reclassifying). If a meme is altered, the original is retained, and a new bi-directional link is created to its new version.

Thus, memes are always preserved as basic information units and building blocks able to be recalled, referenced, combined, and sequenced for any authoring and sharing activity one would like to pursue. However, unlike physical brick and mortar objects or components in an as-built-genealogy of modern supply chain management structures, memes are not expended when used or disbursed. As a virtual agent, their infinite usage potential via associative structural links supports their transdisciplinary employment without setting off unmaintainable attention-consuming redundancy.

## 7. The Negentropic Consequences of the Value-Adding World:3b Services

The supplementary services (Section 6.2.3) aim to effectuate (defined as to put into force or operation) the repository’s affordances to provide further value-adding and negentropic propositions to the PKMS community:

- Metrics: While reputational metrics in social network communities are based on simple clicks, likes, reads, downloads, or responses, academic scholars are accustomed to a sophisticated research and reputation economy where original discoverers/authors are credited based on the evidence provided by citations and references which—weighted by their publisher’s status—contribute to citation indices and impact factors [34]. The underlying methods, however, are “generations old and by now are totally inadequate for their purpose” [22]. Seventy-five years after Bush’s critique, they are still following the book-age paradigm centered on the stable context of far too large paper-or-pdf-based granularities, unable to adequately respond to today’s volatile digital content scenarios by affording effective incentives for wider and faster diffusion and for more rapid iterative improvement. By advancing negentropy, granularity, generativity, and traceability, WHOMER’s metrics afford vital differences.
- Reporting Services: Being at the forefront of transdisciplinary idea generation and innovation, knowledge generation and diffusion can be further enhanced by unearthing promising leads and emerging trends way before link-based search algorithms are able to channel the attention of knowledge workers and entrepreneurs towards exciting new developments.
- Boundary Object Affordances: By bridging disciplinary divides or by aiding transitions from ill-to-well-structured representations (e.g., standards or infrastructure), boundary objects (BO) offer a shared collaborative space of common understanding to afford diverse social actors interpretative and tailorable flexibility to local and disciplinary contexts [38]. The PKMS affordances predestine its application for creating and using BO-artefacts as well as experience management. While the latter relies on “methods and technologies that are suitable for collecting experiences from various sources (documents, data, experts, etc.)”, the former requires identifying and decontextualizing reusable parts of successful practices and solution to favor defined generic viable approaches fitting wider classes of tasks and problem spaces [44]. Such artefacts (e.g heuristics, frameworks, or templates) can be easily shared by the PKMS community members or expertly developed by WHOMER professionals.
- The PKMS Educational Learning Assets Agenda for Personal Learning Environments (PLEs): Irrespective of the remedies put forward in this article, Digital Personal Learning conceptualizations are gaining momentum aiming for designing motivational content and enabling interventions and for the further and faster (self-)development of learners’ potentials [45,46]. As an example of digital platform ecosystems (DPE), collaborating learning management systems (LMS) and knowledge management systems (KMS) are meant, in this respect, to accommodate social actors with highly diverse ambitions and skills as well as expectations to gainfully utilize the DPEs’ resources and generative potential in their personal and local contexts [47]. With the PKMS logic and logistics employed to create LMS-compatible memetic e-learning assets, the learner may also be supplied with their respective PKMS memes for easing retention, referencing, repurposing, and further studying utilizing complementing WHOMER traceabilities. Further value is anticipated in respect to afford the non-linear LMS learning opportunities [48].
- Notifying Services: Having reflected on the merits of attention-guiding/saving reporting to circles of people above, affording the same purposefulness to individualized spaces seems a logical next step. It may, hence, be prudent—within a meme’s genealogical tree or relational network—to notify their linked meme siblings and, by extension, those who employed them about certain state changes of their parent memes (e.g., update or expiry notifications, endorsements, retractions, withdrawals, or falsifications). First-time interests in memes may include informing potential users by simple markers or icons. Once memes have been utilized or cited, authors seldom undertake any post-monitoring or follow-ups which may deprive them from revising or advancing their work; being made explicitly aware of these opportunities can provide a welcome added value.
- Institutional KMS Co-evolutions: LMS and Notifying (personal) versus Metrics and Reporting (public) are services positioned on the opposite ends of a cooperative continuum which also are mirrored in the world:1 technology ecosystem (autonomy versus collaboration) as well as world:2 knowledge worker and society ecosystems. This continuum represents a myriad of externally clustered subsets as well as potential agglomerations where world:2 professionals and their stakeholders may form institutions and where organizational intelligence and memories may need to be captured, synthesized, and protected and the demand for further types of prospective Institutional WHOMER (or I-HOMER) services may originate.

These constructivist features further assist the affordances described earlier for fusing distributed varied sets of virtual memes into memeplexes, knowledge assets, and the unified WHOMER knowledge base with its non-linear associative cohesive information-richness, a missing feature in the still dominating book-age paradigm approaches.

At the grass-roots level, a PKMS user acquires the opportunity for self-reflecting monologues with his/her former states of retainable non-redundant digitalized personal knowledge over lifelong learning and productive periods. This cumulatively synthesized personal extelligence is biographically self-determined, meets today’s increasing demands for the mobility and portability of a knowledge worker’s skills and capacities, and provides the autonomy of how one’s expertise may be shared with personal and professional acquaintances for mutual benefit.

## 8. Discussion: From Entropic to Negentropic States

The contribution aimed for in this, previous and prospective publications is three-fold: 1. To inform relevant stakeholders about the status quo by using a systems thinking and entropic perspective; 2. To convince a progressing part of their critical mass that a viable remedial option exists (as a prerequisite for creating the respective future generative KM reality); 3. To propose the decentralized personal KMS technology which not only details a sustainable solution but—at the same time—brings with it the generative affordances to realize the potential of the distributed talent of knowledge workers.

A PKMS-like technology, in this respect, offers the technological means for instantiating a sustainable ‘nano’-level (of individual actions and contributions) within a four-dimensional nano-micro-meso-macro framework suggested to smooth the process of product/artefact innovation and market shaping: Based on individual knowledge derived from the subjective perceptions/cognition of actors (nano), rules, routines, and catallactic interactions assist in generating novelties in specific contexts (micro) which may (depending on organizational action plans, individuals’ capabilities, and relationships) lead to the formulating of new conceptualizations as well as changes in routinized actions (meso) and to the creation of an artefact and emergence of novel market segments which—in turn—shape the artefact via feedback loops (macro) [49].

A firmly established PKMS may, hence, have the power to transform today’s economies where “every economic actor possesses [only] a certain bit of knowledge specific to himself. [Currently,] knowledge is dispersed and never completely available for everyone; therefore, it is not possible for an individual to know how objects in their environment can be used in different contexts” [49] (p. 7). To assess this likelihood, a recent article [40] argues that, since generative potentials/threats are ignored, dominant KMS designs do currently not yet exist. This inefficient status quo presents the PKMS with a competitive opportunity which has been verified by positively benchmarking its envisaged sustainable impact [3] against twelve objective criteria of disruptive innovations and general-purpose technologies (GPT) [50].

Should a PKMS-like innovation not convince sufficient numbers of adopters, the novel concepts proposed (including the memetic granularity) will fail to counterbalance other emerging entropic granularities which have started dominating the challenging developmental contexts, knowledge workers are already facing, including:

- Current digital technologies are facilitating highly granular networks of instantly, continuously, and ubiquitously connected agents empowered to collaboratively create and directly share information without the need of market intermediaries [51]. They are constrained not only by humans’ finite attention capabilities but also diverse concerns (e.g., confidentiality, copyrights, commercial interests, and market dominance strategies based on service barriers, captured audiences, walled garden approaches) and deficiencies (e.g., incompatibilities, lack of tools and functionalities).
- Flexible labour amounts (rather than discrete units of uniform personbytes) are defining shifting demand patterns altering the granularity of labour markets with transferring the control over when, where, how, and with whom to offer one’s time and competencies to the individual supplier [51], accompanied by rising competitive pressures, further evolving domain-specific knowledge and specializations as well as growing needs for flexible skill sets and self-development [52].
- Current network economies are differentiating between content creation, delivery, and distribution services. The unbundling of the messages from the medium and their re-bundling are propagating snowballing information granularities and entropies of off-the-rack, on-demand, and/or tailor-made output configurations with further content and feedback constantly devised by social media users as well as by platform algorithms based on individuals’ social media preferences [51].
- While social media platforms provide granular forms of voluntary connectivity between personbytes by creating social capital as a kind of digital word-of-mouth communication in neighborhoods and communities, institutional granularity embodies essential firmbyte structures for managing and disintermediating supply, production, and distribution processes across complex value chains as a means for the combinatorial innovation and competitiveness of organizational capital [51]. SECI and Ba concepts [25] explicitly urge leaders to nurture individual autonomy, knowledge-related personal proficiencies/assets, and creative interactions for converting ‘nano’ and personbyte into firmbyte and networkbyte performances. Traditional centralized top-down approaches, nevertheless, still neglect synergies to collectively benefit from (personal and organizational) prior accumulated knowledge subsets, absorptive capacities, ambidextrous and dynamic capabilities at the expense of disinterested employees and failed KMS approaches.

## 9. Conclusions and the Road Ahead

A flourishing PKMS-like roll-out, on the other hand, affords tackling these unsustainable scenarios by delivering towards the rich host of potential synergies alluded to and, hence, foster enabling ‘ba’ environments for a prospective prospering PKMS community engaged in KM and DSR endeavors. Creativity and innovativeness benefit from PKMSs affording cumulative synthesis [31] and attention management. As reasoned by Simon five decades ago [2], progress in a knowledge-rich world depends on extracting the redundancy (entropy) and strengthening and exploiting the patterns (negentropy) of the world, so that far less information needs to be read, written, or stored.

In these contexts, PKMSs are “aiming for novel sustainable interventions to confront opportunity divides by affording individuals the means for life-long-learning, resourcefulness, creative authorship and teamwork and by supporting their generative role as contributor to and beneficiary of organizational and societal performances” [40] (p. 13) They, hence, yield strong synergies with their traditional top-down-KMS-correspondents allowing for collaboratively interlinking knowledge bases and for collectively tracing, harvesting, and utilizing accumulated knowledge subsets more productively for organizational as well as personal benefit.

The bottom-up-PKMS approach is firmly rooted in the PKMS’s granular memetic record structure and the interdisciplinary classification and curation system of the WHOMER repository, which not only allows for effectively combining individual ‘nano’ actions and contributions but also provides for an interrogatable tangible Popperian world:3 with its diffusible content in non-linear transdisciplinary and learning contexts.

A PKMS e-learning agenda [48] and a PKM for Development (PKM4D) framework [53] guides accessing content and devices, furthering individual proficiencies, facilitating collaborations, contributing to the world’s record, and aiding self-transcendence while ensuring individuals’ attention preservation, knowledge retention, and privacy protection.

Initially provoked by the lately evolving entropic and unsustainable states alluded to, the design of the proposed innovative remedies (PKMS with its SICEE workflows) follows the nano-micro-meso-macro-and-feedback reasoning (applying its logic and logistics by storing all related publications in their meme-based representations for PKMS testing). It reflects on expected shifting and/or rising market demands (GPTs and the significance of dominant designs) as well as on recently introduced enabling technologies (e.g., cloud-computing, rapid development platforms, and noSQL-databases).

Further publications and posters are under review or planned to address a PKMS sustainability vision in the form of a case study considering how the memetic PKMS storage compares to traditional document-centric approaches (e.g., Google Scholar, ResearchGate), and how the PKMS concept compares to, can make use of, and add to semantic web technologies. After completing the test phase of the prototype, its transformation into a viable PKMS device application is estimated to take 12 months.

## Figures and Tables

**Table 1:** Identified Causes and positive/negative Effects segmented according to knowledge clusters.

	Public Knowledge	Private Knowledge
**Discoverable Knowledge**	Information Entropy [1]	Online and Publishing Realities [2]
	Information Overload (-)	More rapid iterative Improvement (+)
	Attention Poverty, Mobility (-)	Innovation & Reputation Systems (+)
**Undiscoverable Knowledge**	Structural Holes, Islands, Siloes [3]	Invisible Work, Scaffolding [6]
	Ineffective Utilization [4]	Non-Linear Relationships [7]
	Deficient Awareness/Education [5]	Unproductive Rework (-)
	Innovation and Opportunity Divides (-)	Holistic Understanding (-)
